# Pre-existing cardiovascular disease rather than cardiovascular risk factors drives mortality in COVID-19

**DOI:** 10.1186/s12872-021-02137-9

**Published:** 2021-07-03

**Authors:** Kevin O’Gallagher, Anthony Shek, Daniel M. Bean, Rebecca Bendayan, Alexandros Papachristidis, James T. H. Teo, Richard J. B. Dobson, Ajay M. Shah, Rosita Zakeri

**Affiliations:** 1 Department of Cardiology, King’s College London British Heart Foundation Centre of Research Excellence, School of Cardiovascular Medicine and Sciences, London, UK; 2 Department of Biostatistics and Health Informatics, Institute of Psychiatry, Psychology and Neuroscience, King’s College London, London, UK; 3 NIHR Biomedical Research Centre at South London and Maudsley NHS Foundation Trust and King’s College London, London, UK; 4 King’s College Hospital NHS Foundation Trust, London, UK; 5 Health Data Research UK London, Institute of Health Informatics, University College London, London, UK; 6 School of Cardiovascular Medicine and Sciences, James Black Centre, King’s College London, 125 Coldharbour Lane, London, SE5 9NU UK

**Keywords:** COVID-19, Cardiovascular disease, Cardiovascular risk factors, Hypertension, Diabetes

## Abstract

**Background:**

The relative association between cardiovascular (CV) risk factors, such as diabetes and hypertension, established CV disease (CVD), and susceptibility to CV complications or mortality in COVID-19 remains unclear.

**Methods:**

We conducted a cohort study of consecutive adults hospitalised for severe COVID-19 between 1st March and 30th June 2020. Pre-existing CVD, CV risk factors and associations with mortality and CV complications were ascertained.

**Results:**

Among 1721 patients (median age 71 years, 57% male), 349 (20.3%) had pre-existing CVD (CVD), 888 (51.6%) had CV risk factors without CVD (RF-CVD), 484 (28.1%) had neither. Patients with CVD were older with a higher burden of non-CV comorbidities. During follow-up, 438 (25.5%) patients died: 37% with CVD, 25.7% with RF-CVD and 16.5% with neither. CVD was independently associated with in-hospital mortality among patients < 70 years of age (adjusted HR 2.43 [95% CI 1.16–5.07]), but not in those ≥ 70 years (aHR 1.14 [95% CI 0.77–1.69]). RF-CVD were not independently associated with mortality in either age group (< 70 y aHR 1.21 [95% CI 0.72–2.01], ≥ 70 y aHR 1.07 [95% CI 0.76–1.52]). Most CV complications occurred in patients with CVD (66%) versus RF-CVD (17%) or neither (11%; *p* < 0.001). 213 [12.4%] patients developed venous thromboembolism (VTE). CVD was not an independent predictor of VTE.

**Conclusions:**

In patients hospitalised with COVID-19, pre-existing established CVD appears to be a more important contributor to mortality than CV risk factors in the absence of CVD. CVD-related hazard may be mediated, in part, by new CV complications. Optimal care and vigilance for destabilised CVD are essential in this patient group. *Trial registration* n/a.

**Supplementary Information:**

The online version contains supplementary material available at 10.1186/s12872-021-02137-9.

## Background

Cardiovascular (CV) risk factors such as hypertension and diabetes, and chronic CV diseases (CVD), including ischaemic heart disease and heart failure, are highly prevalent among patients admitted to hospital with severe novel coronavirus disease 2019 (COVID-19) [1–5]. In population-based studies, diabetes and chronic CVD, but not hypertension, have been associated with higher mortality [6, 7]. At present, patients with either established CVD or CV risk factors are considered to be vulnerable individuals [8]. However, it remains unclear whether an increased susceptibility to severe COVID-19 in patients with CV risk factors is driven by co-existent CVD, or whether patients with CV risk factors without established CVD have a similarly severe course.

The relationship between CVD and COVID-19 may also be bidirectional. SARS-CoV-2 is reported to directly infect the endothelium and possibly the heart [9, 10], which could precipitate CV complications. Isolated case reports of fulminant myocarditis or pericarditis have been attributed to COVID-19 [11–13], although the incidence and mechanism of such complications is debated. Furthermore, while patients with pre-existing CVD may be at increased risk of CV complications [14, 15], it is not clear the extent to which these represent recurrent or decompensated CVD rather than de novo complications, nor whether the risk also applies to patients with CV risk factors.

To address these questions, we evaluated outcomes associated with pre-existing CVD and CV risk factors, in a large multi-ethnic cohort of patients hospitalised for severe COVID-19. Our aims were to determine (a) the relative risks of in-hospital mortality and CV complications for individuals with COVID-19 and pre-existing CVD versus CV risk factors without established CVD, and (b) factors associated with the occurrence of major CV complications in patients with COVID-19.

## Methods

### Approvals

This study was conducted under London South East Research Ethics Committee approval (reference 18/LO/2048) granted to the King’s Electronic Records Research Interface (KERRI); COVID-19 work was reviewed with expert patient input on a virtual committee with Caldicott Guardian oversight.

### Study design

We conducted a cohort study of consecutive adult patients (age > 18 y) admitted with COVID-19 to King’s College Hospital NHS Foundation Trust (comprising King’s College Hospital and Princess Royal University Hospital), between 1st March and 30th June 2020. All patients had a positive RT-PCR antigen test for SARS-CoV-2. Only patients admitted to hospital for ≥ 24 h were included. A subset of this cohort has been reported previously [3, 16].

### Data sources and processing

Structured and unstructured data were extracted from the electronic health record (EHR) using previously described natural language processing (NLP) informatics tools belonging to the CogStack ecosystem [17], DrugPipeline [18], MedCAT [19], and MedCATtrainer [20]. Clinician case review was used for additional validation (Additional file 1: Methods).

### Exposures and outcomes

CV risk factors were defined as a recorded clinical diagnosis of hypertension, diabetes mellitus, or self-reported smoking status, in the absence of documented CVD. Diabetes mellitus was defined by a clinician diagnosis documented in the electronic health record and extracted based on relevant SNOMED CT UK extension and children terms (S-44054006, S-73211009, S-46635009) encompassing both type 1 and type 2 diabetes mellitus, as previously described [19] and validated [16]. Pre-existing established CVD was defined as a previous record of ≥ 1 of the following diagnoses: myocardial infarction (MI), heart failure, myocarditis, pericarditis, endocarditis, atrial or ventricular arrhythmia, and severe valvular heart disease [21]. Additional details are provided in Additional file 1: Methods. CV risk factors and pre-existing CVD were categorised as present if they had been recorded in the EHR at any time up to the day of admission (or including the day of admission, when recorded as a pre-existing condition). Data were also collected for age, sex, ethnicity, body mass index (BMI), non-CV comorbidities (asthma, chronic obstructive pulmonary disease [COPD], chronic kidney disease [CKD]), previous venous thromboembolism [VTE] comprising deep vein thrombosis [DVT] or pulmonary embolism [PE]), CV drug therapy (Additional file 1: Methods) and clinical examination and routinely collected blood results on admission. High sensitivity cardiac troponin T (hs-cTnT) plasma levels were defined as normal when below the 99th percentile of normal values, i.e., 14 ng/L.

The primary outcome was in-hospital mortality, with cause of death ascertained from death certification. Secondary outcomes included any CV complication related to COVID-19 and incident VTE. A CV complication was defined as a new CVD diagnosis or decompensation of pre-existing CVD recorded in the EHR on presentation or at any time during admission. CV complications were based on clinician diagnoses incorporating all available clinical information, including where relevant, echocardiography and coronary angiography (additional details in Additional file 1: Methods). Hospital admission date was used as the start of follow-up. Outcomes were ascertained through to death, discharge, or 31st July 2020, whichever was earlier.

### Statistical analyses

Patient data are reported as frequency (%), mean (SD) or median (IQR), as appropriate. Patient characteristics were compared across 3 groups: patients with pre-existing established CVD (CVD), CV risk factors without CVD (RF-CVD), and no CVD or CV risk factors, using the Chi-squared goodness of fit or Fisher’s exact test (categorical variables), one-way analysis of variance (continuous variables) or Kruskal–Wallis/Wilcoxon rank-sum tests (for non-normally distributed data). Bonferroni correction was used for individual comparisons. Missing blood biomarkers (< 25% missing data) were imputed using the multiple imputation approach by chain equations [22].

Cumulative incidence plots displaying the probability of in-hospital mortality and discharge were constructed based on a competing risks analysis. To evaluate the association between patient group and mortality, we used Cox proportional hazards regression, with admission date as the start of follow-up and in-hospital mortality as the dependent variable. Unadjusted, demographic adjusted (age, sex, ethnicity), and fully adjusted models (including non-CV comorbidities and medications on admission) were performed. Age was modelled as a categorical variable to allow for potentially non-linear association (< 40, 40–49, 50–59, 60–69, 70–79, 80+ years). Comorbidities were modelled as binary variables. The reference group comprised patients without pre-existing CVD or CV risk factors. The proportional hazard assumption was examined graphically and using formal tests, as described by Grambsch [23]; no major deviations from this assumption were observed.

To investigate the association between CV complications and prognosis we performed logistic regression models with patient groups stratified by the presence or absence of CV complications as an independent categorical variable. For secondary outcomes of CV complications or VTE, logistic regression models were constructed with (1) patient group and (2) individual CV risk factors or CVDs as binary predictor variables. Unadjusted, demographic-adjusted, and fully adjusted regression models were performed as above. When individual CVDs were examined, myocarditis and pericarditis were excluded, due to their low prevalence. Information regarding hyperlipidaemia diagnoses were incomplete. A sensitivity analysis was performed where all patients who were prescribed statin therapy in the absence of diagnosed CVD were reclassified as RF-CVD. Furthermore, as BMI was missing in > 30% of patients, primary analyses were performed without adjustment for BMI. However, sensitivity analyses were performed restricted to patients with BMI data available including (1) adjustment for BMI as a continuous variable (fully adjusted model), (2) adjustment for obesity as a categorical variable (defined as BMI ≥ 30 kg/m^2^), and (3) reclassifying patients with obesity without diagnosed CVD as RF-CVD. An additional sensitivity analysis was performed among individuals with outcome data available i.e. those who were discharged or died (excluding current in-patients). Analyses were performed using STATA/IC (v16.1; StataCorp LLC, TX).

## Results

### Study population

Between 1st March and 30th June 2020, 1,721 patients were admitted with COVID-19 (median age 71 years [IQR 56–83], 56.6% male). Of these, 349 (20.3%) had CVD, 888 (51.6%) had RF-CVD, and 484 (28.1%) had neither. Patients with CVD were older than patients with RF-CVD or neither but had a similar distribution of sex (Table 1).

**Table 1:** Patient characteristics

	Total N = 1721	No CVD or CV risk factors N = 484 (28.1%)	RF-CVD N = 888 (51.6%)	CVD N = 349 (20.3%)	*p* value
*Demographics*					
Age, y	71 (56–83)	58 (44–75)	71 (59–82)	81 (71–88)	< 0.001
*Age group, n (%)*					< 0.001
< 40	132 (7.7)	97 (20.0)	31 (3.5)	4 (1.2)	
40–49	127 (7.4)	64 (13.2)	55 (6.2)	8 (2.3)	
50–59	266 (15.5)	93 (19.2)	149 (16.8)	24 (6.9)	
60–69	303 (17.6)	73 (15.1)	187 (21.1)	43 (12.3)	
70–79	316 (18.4)	56 (11.6)	173 (19.5)	87 (24.9)	
80+	577 (33.5)	101 (20.9)	293 (33.0)	183 (52.4)	
Male sex	974 (56.6)	259 (53.5)	522 (58.8)	193 (55.3)	0.146
BMI, kg/m^2^	26.2 (22.5–31.1)	25.4 (21.8–30.6)	26.9 (23.1–31.8)	25.1 (22.0–29.0)	< 0.001
BMI category*					< 0.001
Underweight	74 (4.3)	23 (4.8)	29 (3.3)	22 (6.3)	
Normal weight	349 (20.3)	103 (21.3)	152 (17.1)	94 (26.9)	
Overweight	291 (16.9)	71 (14.7)	155 (17.5)	65 (18.6)	
Obese	317 (18.4)	83 (17.2)	177 (19.9)	57 (16.3)	
Missing	690 (40.1)	204 (42.2)	375 (42.2)	111 (31.8)	
*Ethnicity*					< 0.001
White	845 (49.1)	238 (49.2)	382 (43.0)	225 (64.5)	
Black	434 (25.2)	85 (17.6)	280 (31.5)	69 (19.8)	
Asian	96 (5.6)	29 (6.0)	55 (6.2)	12 (3.4)	
Mixed/other	121 (7.0)	37 (7.6)	64 (7.2)	20 (5.7)	
Missing	225 (13.1)	95 (19.6)	107 (12.1)	23 (6.6)	
*Comorbidities*					
Cardiovascular risk factors					
Hypertension	963 (56.0)	–	689 (77.6)	274 (78.5)	0.726
Diabetes	601 (34.9)	–	440 (49.6)	161 (46.1)	1.000
Type 1	6 (0.4)	–	5 (0.6)	1 (0.3)	0.305
Type 2	595 (34.6)	–	435 (49.0)	160 (45.9)	0.320
Ever smoker	432 (25.1)	–	314 (35.4)	118 (33.8)	0.607
Current smoker	114 (6.6)	–	85 (9.6)	29 (8.3)	0.490
Ex-smoker	318 (18.5)	–	229 (25.8)	89 (25.5)	0.917
Cardiovascular diseases					
Previous myocardial infarction	107 (6.2)	–	–	107 (30.7)	–
Chronic heart failure	133 (7.7)	–	–	133 (38.1)	–
Previous myocarditis	4 (0.2)	–	–	4 (1.2)	–
Previous pericarditis	3 (0.2)	–	–	3 (0.9)	–
Arrhythmia	218 (12.7)	–	–	218 (62.5)	–
Atrial fibrillation	188 (10.9)	–	–	188 (53.9)	–
Valvular heart disease**	15 (0.9)	–	–	15 (4.3)	–
Previous endocarditis	16 (0.9)	–	–	16 (4.6)	–
Non-cardiac comorbidities					
Asthma	148 (8.6)	16 (3.3)	74 (8.3)	58 (16.6)	< 0.001
COPD	129 (7.5)	5 (1.0)	53 (6.0)	71 (20.3)	< 0.001
Chronic kidney disease	165 (9.6)	6 (1.2)	71 (8.0)	88 (25.2)	< 0.001
Previous pulmonary embolism	90 (5.2)	8 (1.7)	27 (3.0)	55 (15.8)	< 0.001
Previous deep vein thrombosis	110 (6.4)	12 (2.5)	37 (4.2)	61 (17.5)	< 0.001
*Medication*					
ACEI/ARB	528 (31.4)	29 (6.5)	353 (39.9)	146 (41.8)	< 0.001
Aldosterone antagonist	66 (3.9)	11 (2.5)	24 (2.7)	31 (8.9)	< 0.001
Beta-blocker	430 (25.6)	43 (9.6)	197 (22.3)	190 (54.4)	< 0.001
Calcium-channel blocker	458 (27.3)	26 (5.8)	345 (39.0)	87 (24.9)	< 0.001
Loop diuretic	245 (14.6)	20 (4.5)	93 (10.5)	132 (37.8)	< 0.001
Statin	678 (40.3)	64 (14.3)	420 (47.5)	194 (55.6)	< 0.001
Anticoagulant	325 (19.3)	53 (11.8)	108 (12.2)	164 (47.0)	< 0.001
Antiplatelet agent	395 (23.5)	47 (10.5)	222 (25.1)	126 (36.1)	< 0.001
Metformin	299 (17.4)	–	240 (27.0)	59 (16.9)	–
Sulphonylurea	128 (7.4)	–	104 (11.7)	24 (6.9)	–
Repaglinide	–	–	–	–	–
SGLT2 inhibitor	23 (1.3)	–	17 (1.9)	6 (1.7)	–
DPP4 inhibitor	134 (7.8)	–	88 (9.9)	46 (13.2)	–
Thiazolidinedione	4 (0.2)	–	3 (0.3)	1 (0.3)	–
GLP1 receptor agonist	14 (0.8)	–	8 (0.9)	6 (1.7)	–
Insulin	180 (10.5)	–	128 (14.4)	52 (14.9)	–
*COVID-19 investigational therapies*					
Hydroxychloroquine	15 (0.9)	6 (1.2)	7 (0.8)	2 (0.6)	0.553
Dexamethasone/Prednisolone	204 (11.9)	50 (10.3)	103 (11.6)	51 (14.6)	0.159
Remdesivir	2 (0.1)	1 (0.2)	–	1 (0.3)	–
Colchicine	16 (0.9)	2 (0.4)	7 (0.8)	7 (2.0)	0.049
Tocilizumab	2 (0.1)	–	–	2 (0.6)	–
Azithromycin	26 (1.5)	13 (2.7)	8 (0.9)	5 (1.4)	0.035

Data represent n (%) or median (IQR)

*p* values refer to comparisons across 3 groups (except for cardiovascular risk factors, where comparisons are between 2 groups: CVD vs. RF-CVD)

*ACEI* angiotensin converting enzyme inhibitors, *ARB* angiotensin receptor blockers, *BMI* body mass index, *COPD* chronic obstructive pulmonary disease, *CV* cardiovascular, *CVD* cardiovascular disease, *DPP4* dipeptidyl peptidase-4, *GLP1* glucagon-like peptide 1,  *RF-CVD* cardiovascular risk factors without established CVD, *SGLT2* sodium glucose co-transporter-2

*BMI categories classified as: underweight (< 18.5 kg/m^2^), normal (18.5–24.9 kg/m^2^), overweight (25–29.9 kg/m^2^), and obese (≥ 30 kg/m^2^)

**Severe degree of valvular heart disease (ESC guidelines [21])

CVD was more prevalent with increasing age, while RF-CVD was most common between 50–70 years (Additional file 3: Fig. S1A). Individuals from non-White ethnic groups had a higher prevalence of RF-CVD whereas CVD was more prevalent in the White group (Additional file 3: Fig. S1B). The most frequent CVD diagnoses were arrhythmia (86.2% atrial fibrillation), heart failure, and previous MI, respectively (Table 1). 119 (34.1%) patients with CVD had more than one CVD diagnosis. Rates of non-cardiovascular comorbidities were highest in patients with CVD, followed by RF-CVD (Table 1).

On admission, 83% of patients with hypertension were taking an antihypertensive agent and 73% of patients with atrial fibrillation were on oral anticoagulation. Rates of ACEI or ARB and betablocker use in heart failure patients were 47% and 62% respectively. In patients with a previous MI, rates of antiplatelet, beta-blocker and statin use were 68%, 64% and 65% respectively.

### Clinical presentation

Physiological parameters and blood biomarkers are displayed in Additional file 2: Table S1. There were few clinically significant differences in physiological observations between groups, with the exception of a higher mean systolic blood pressure in patients with RF-CVD. Among blood biomarkers, C-reactive protein values were highest in patients with RF-CVD, but similar between patients with CVD and the group with neither CVD nor risk factors (Additional file 2: Table S1). Renal function was progressively worse across groups, with the lowest eGFR in patients with CVD.

Overall, 742 (43.1%) patients had at least one hs-cTnT measurement. Among patients with at least one hs-cTnT measurement, elevated values (> 14 ng/L) were observed in 133/147 (90.5%) patients with CVD, 311/409 (76.0%) patients with RF-CVD and 123/186 (66.1%) patients with no CVD or CV risk factors.

### In-hospital mortality

In-hospital outcomes are displayed in Table 2. Overall, 438 (25.5%) patients died and 1246 (72.4%) were discharged alive. 37 (2.1%) patients were in hospital at study close. The median length of hospitalisation for patients discharged was 9 (IQR 4–17) days and was longer for patients with CVD than those without (11 [IQR 5–19] vs. 7 [IQR 3–16] days, *p* < 0.001). Among patients who died, finalised death certificates were available in 382 patients. COVID-19 related pneumonia or acute respiratory distress syndrome was reported as the direct cause of death in 302 (79.1%) patients and as an indirect cause of death in 68 (17.8%) patients due to complications associated with COVID-19. This included 20 patients who died due to a CV cause: stroke (n = 8), massive PE (n = 3), decompensated heart failure (n = 4), myocardial infarction (n = 4) and acute limb ischaemia (n = 1). Only 12 deaths (3.1%) deaths were not attributed to COVID-19, e.g., malignancy or advanced dementia.

**Table 2:** Complications and in-hospital outcomes of patients with COVID-19

	Total N = 1721	No CVD or CV risk factors N = 484 (28.1%)	RF-CVD N = 888 (51.6%)	CVD N = 349 (20.3%)	*p* value
*Complications*					
Cardiac					
Acute myocardial infarction	68 (4.0)	4 (0.8)	21 (2.4)	43 (12.3)	< 0.001
Acute heart failure	151 (8.8)	12 (2.5)	43 (4.8)	96 (27.5)	< 0.001
Myocarditis	12 (0.7)	0	9 (1.0)	3 (0.9)	0.090
Pericarditis	2 (0.1)	1 (0.2)	1 (0.1)	0	0.688
Arrhythmia*	314 (18.3)	40 (8.3)	99 (11.2)	175 (50.1)	< 0.001
Atrial fibrillation	266 (15.5)	28 (5.8)	74 (8.3)	164 (47.0)	< 0.001
Number of cardiac complications**					< 0.001
0	1,290 (75.0)	433 (89.5)	738 (83.1)	119 (34.1)	
1	325 (18.9)	45 (9.3)	129 (14.5)	151 (43.3)	
2+	106 (6.2)	6 (1.2)	21 (2.4)	79 (22.6)	
Venous thromboembolism					
Pulmonary embolism	151 (8.8)	41 (8.5)	66 (7.4)	44 (12.6)	0.015
Deep vein thrombosis	98 (5.7)	21 (4.3)	43 (4.8)	34 (9.7)	0.001
Extra-cardiac					
Acute kidney injury*** ARDS Mechanical ventilation	266 (15.5) 77 (4.5) 92 (34.9)	30 (6.2) 19 (3.9) 36 (43.9)	164 (18.5) 46 (5.2) 41 (27.2)	72 (20.6) 12 (3.4) 15 (48.4)	< 0.001 0.324 0.009
*Outcomes*					
Died in hospital	438 (25.5)	80 (16.5)	228 (25.7)	130 (37.3)	< 0.001
ICU admission	226 (13.1)	75 (15.3)	127 (14.3)	24 (6.9)	< 0.001
Death or ICU admission	587 (34.1)	133 (27.4)	311 (35.0)	145 (41.4)	< 0.001
Discharged from hospital alive	1,246 (72.4)	393 (81.2)	639 (72.0)	214 (61.3)	< 0.001
Hospital length of stay^$^, days	9 (4–17)	7 (3–16)	8 (4–18)	11 (5–19)	< 0.001

Data presented as n (%) or median (IQR). Table includes all in-hospital diagnoses during the admission (including new and recurrent diagnoses)

*Any physician-identified cardiac arrhythmia

**Number of physician-diagnosed CV complications from the following: acute myocardial infarction, heart failure, myocarditis, pericarditis, arrhythmia including AF, and endocarditis

***Acute kidney injury was defined according to the Kidney Disease: Improving Global Outcomes definition[39]

^$^Among patients discharged[40–43]

**Fig. 1:**
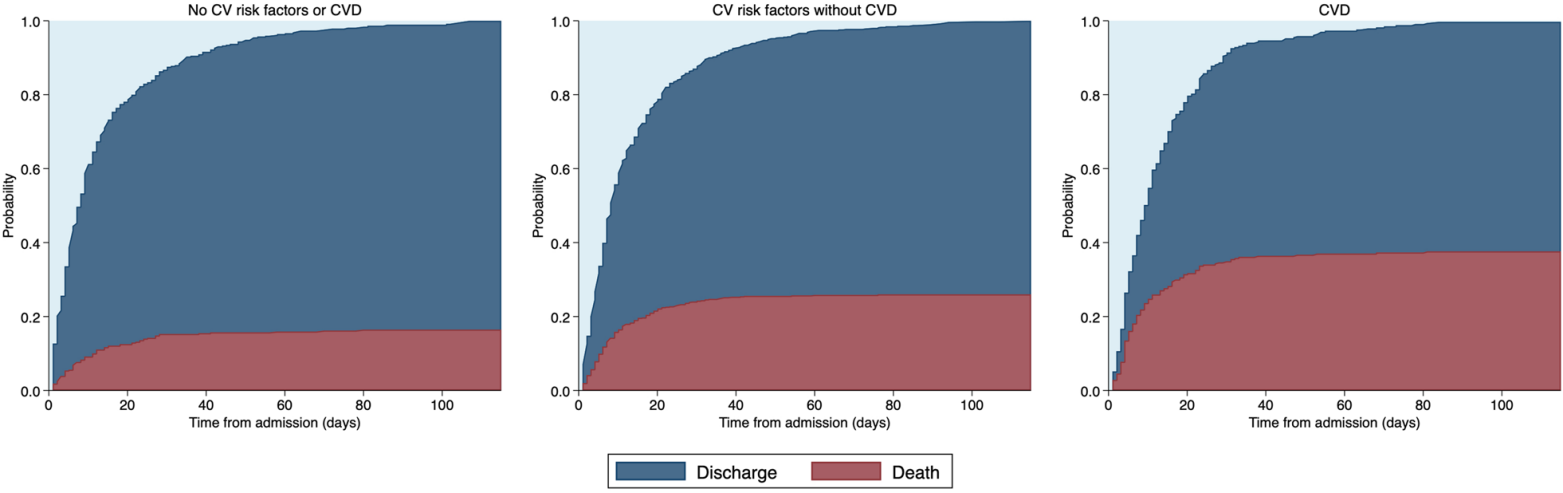
Cumulative incidence plots displaying the probability of in-hospital death and discharge over time. The light blue region represents the probability of being alive and still in hospital at the time of study close. CV denotes cardiovascular; CVD, cardiovascular disease

**Fig. 2:**
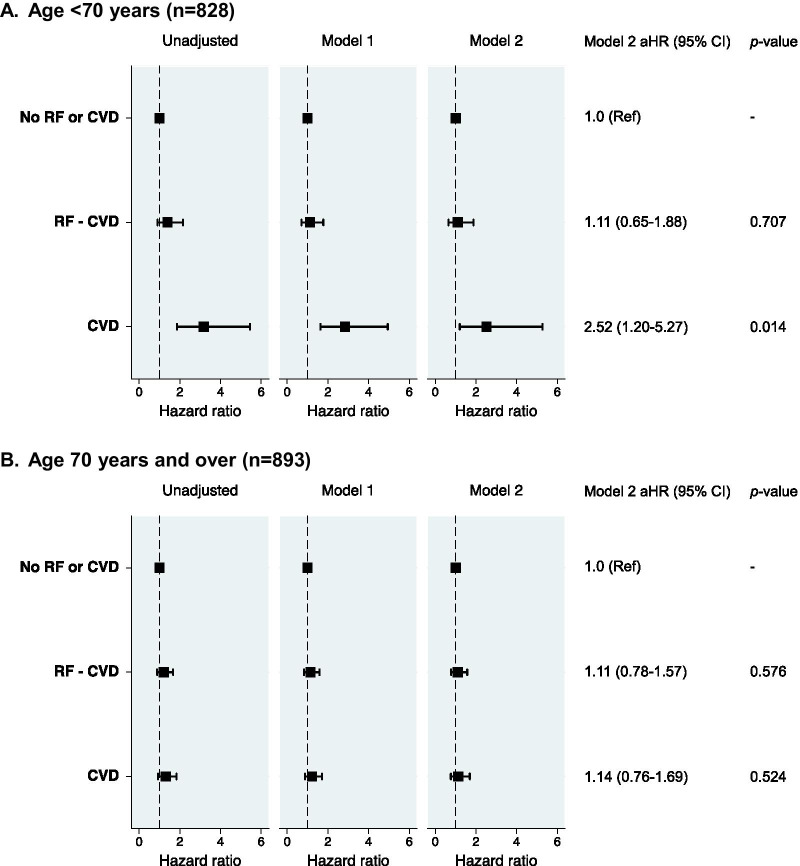
Risk of in-hospital mortality stratified by age and cardiovascular risk group. **A** Age < 70 years (n = 828). **B**. Age 70 years and over (n = 893). aHR denotes adjusted hazard ratio; CVD, cardiovascular disease; RF, cardiovascular risk factors; RF-CVD, cardiovascular risk factors without established CVD. Model 1 adjusted for age, sex and ethnicity. Model 2 adjusted for age, sex, ethnicity, non-cardiac comorbidities (asthma, COPD, chronic renal failure, pulmonary embolism, DVT) and medications (ACEI or ARB, aldosterone receptor antagonists, beta-blockers, calcium channel blockers, loop diuretics, statins, anticoagulants, antiplatelet agents, metformin, sulphonylureas, SGLT2 inhibitors, DPP4 inhibitors, thiazolidinediones, GLP1 receptor agonists, insulin)

In-hospital mortality was greatest among patients with CVD (37.3%), intermediate in patients with RF-CVD (25.7%), and lowest among patients with neither (16.5%). Figure 1 displays cumulative incidence plots of the probability of in-hospital death or discharge over time for each group. For the overall cohort, there was a positive association between CVD and in-hospital mortality in unadjusted (HR 2.17 [95% CI 1.64–2.87], *p* < 0.001), and demographic-adjusted models (adjusted HR 1.45 [95% CI 1.09–1.94], *p* = 0.012). In fully adjusted models additionally accounting for non-CV comorbidities and baseline medications, there was a positive trend (aHR 1.36 [95% CI 0.97–1.92], *p* = 0.076). This effect was principally driven by a prognostic association in patients under 70 years of age (Fig. 2A), whereas the effect of CVD was smaller and non-statistically significant among patients aged 70 years and older (Fig. 2B). RF-CVD conferred an increased risk of mortality for the overall cohort in unadjusted analyses (HR 1.51 [95% CI 1.17–1.95], *p* = 0.002), but not in demographic-adjusted (aHR 1.17 [95% CI 0.90–1.53], *p* = 0.233) or fully adjusted models (aHR 1.13 [95% CI 0.85–1.51], *p* = 0.388). RF-CVD were not associated with mortality in patients older or younger than 70 years of age (Fig. 2A, B).

The main findings were unchanged in a sensitivity analysis with patients prescribed statin therapy without diagnosed CVD reclassified as RF-CVD (n = 64 patients reclassified, Additional file 3: Fig. S2A, B). BMI data were available in 1031 patients (60% total cohort). Sensitivity analyses in this subset adjusting for BMI as a continuous variable, (Additional file 3: Fig. S3A, B) or obesity as a categorical variable (Additional file 3: Fig. S4A, B) demonstrated similar effects to the main analysis. In addition, when obesity was included as a CV risk factor, 83 individuals with a BMI ≥ 30 kg/m^2^ were reclassified as RF-CVD (from the no RF or CVD category). Effect estimates remained robust with only marginally wider confidence intervals (Additional file 3: Fig. S5A, B). Finally, a sensitivity analysis excluding current in-patients also showed similar findings to the main analysis. (Additional file 3: Fig. S6A, B).

### Cardiovascular complications

Cardiovascular complications occurred in 431 (25.0%) patients, with two-thirds occurring in patients with CVD (n = 230, 65.9%). Patients with RF-CVD also had a higher CV complication rate than patients with neither CVD or CV risk factors (16.9% vs. 10.5%, Bonferroni adjusted *p* < 0.001). The most frequent CV complications were cardiac arrhythmias (84.7% atrial fibrillation), followed by acute heart failure (distinct from myocarditis) and acute MI, respectively (Table 2). Among patients presenting with an acute MI, 3 patients displayed ST elevation MI (STEMI) and underwent emergency percutaneous coronary intervention. Two additional patients underwent coronary angiography, one patient was diagnosed with spontaneous coronary artery dissection and one patient diagnosed with myocarditis. The remaining cases of acute MI were clinically considered to represent non-ST elevation or type 2 MIs [24]. The incidence of clinician-diagnosed myocarditis was low (0.7%). When arrhythmia-related complications were excluded, 59% of complications occurred in patients with CVD, 33% in patients with RF-CVD, and 8% in patients with neither.

**Fig. 3:**
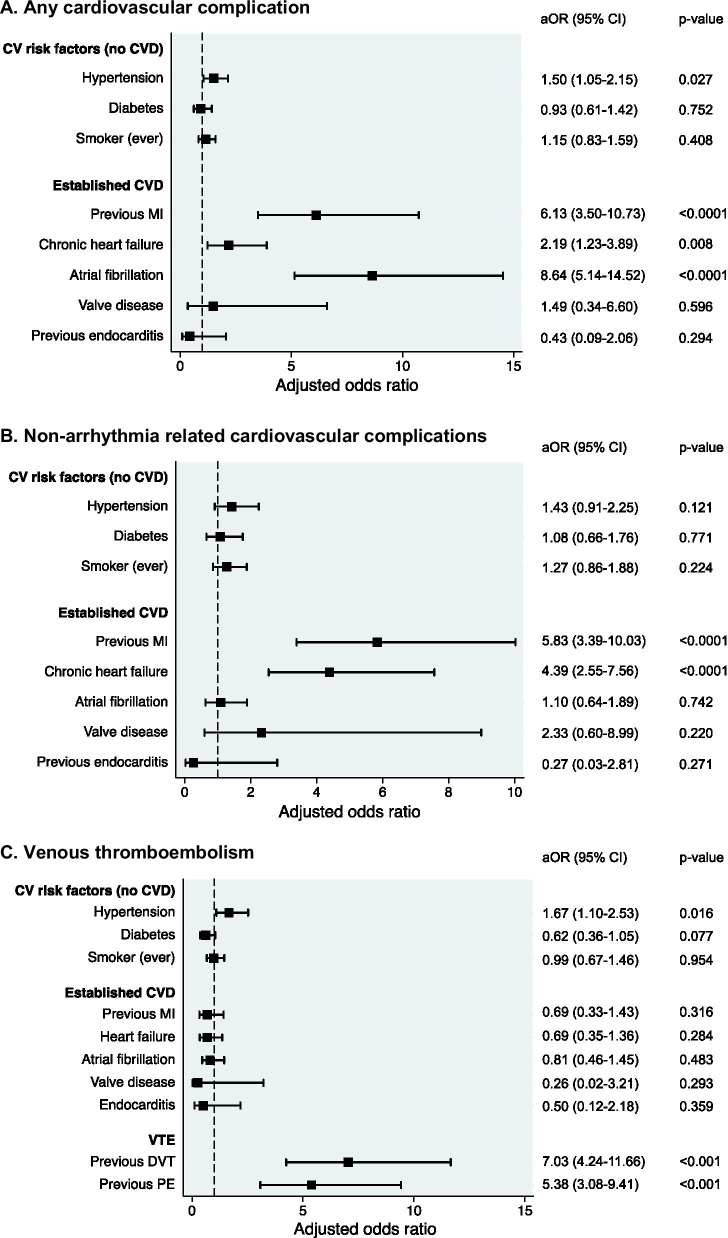
Risk of COVID-19 related complications by cardiovascular risk group. **A** Any cardiovascular complications. **B** Non-arrhythmia related cardiovascular complications. **C** Venous thromboembolism. aOR, adjusted odds ratio; CV, cardiovascular; CVD, cardiovascular disease; DVT, deep vein thrombosis; PE, pulmonary embolism; MI, myocardial infarction; VTE, venous thromboembolism. Each model is adjusted for the variables listed, as well as age, sex, ethnicity, non-cardiac comorbidities (asthma, COPD, chronic renal failure, pulmonary embolism, DVT) and medications (ACEI or ARB, aldosterone receptor antagonists, beta-blockers, calcium channel blockers, loop diuretics, statins, anticoagulants, antiplatelet agents, metformin, sulphonylureas, SGLT2 inhibitors, DPP4 inhibitors, thiazolidinediones, GLP1 receptor agonists, insulin)

In patients with CVD, the majority of CV complications represented exacerbations or decompensation of underlying CVD, rather than a new presentation, e.g., 86% of myocardial infarctions occurred in individuals with a previous myocardial infarction (Additional file 3: Fig. S7). Among specific CVDs and risk factors, pre-existing AF was associated with the highest adjusted odds of having any CV complication, followed by previous MI (Fig. 3A). For non-arrhythmia related CV complications, the highest adjusted odds were seen in patients with a previous myocardial infarction (Fig. 3B).

When CV complications were defined by cardiac biomarker elevation, in the subset of patients with a hs-cTnT measurement (n = 742), the presence of troponin elevation (Additional file 3: Fig. S8A) or troponin-elevation greater than 10 × normal (Additional file 3: Fig. S8B) were both associated with increased odds of in-hospital mortality across groups.

The incidence of VTE was higher in patients with CVD versus RF-CVD or neither (18.3% vs. 10.9% and 10.7% respectively, *p* < 0.001 for each). However, among CVDs and CV risk factors, hypertension (in the absence of established CVD) was the only independent CV predictor of VTE. Patients with previous VTE (n = 166, 48% anticoagulated at admission) had the highest rate of new (incident) VTE (49.4% vs. 8.4% with no previous VTE *p* < 0.001), and previous VTE was the strongest predictor of incident VTE, including adjustment for baseline anticoagulation use (Fig. 3C).

## Discussion

We investigated the inter-relationship between CVD, CV risk factors, CV complications and mortality among 1721 consecutive patients hospitalised due to COVID-19. Overall, 20% of the cohort had CVD and an additional 50% had CV risk factors without yet having developed CVD (RF-CVD). A major finding is that the age- and sex-adjusted mortality risk is markedly increased in patients aged under 70 years with CVD but is only modestly and non-significantly increased in those with RF-CVD. The mortality risk associated with CVD appears much lower in individuals above 70 years of age. We also found that 1 in 4 patients hospitalised with COVID-19 experienced a CV complication, with cardiac arrhythmias representing the most common diagnosis, and the majority of CV complications and myocardial injury occurred in patients with CVD. Myocardial injury as indicated by an elevated troponin level was an independent predictor of mortality. Taken together, these findings suggest that pre-existing established CVD rather than CV risk factors per se influence mortality in severe COVID-19 and that this effect may be driven at least in part by CV complications and injury.

### Pre-existing cardiovascular disease

The prevalence of CVD in our study was similar to other large hospital cohorts [4, 25]. We corroborate previous reports showing that a history of CVD is associated with greater risk of COVID-19-related mortality [4–6]. Interestingly, we identify an interaction with age, wherein the increased mortality risk is mainly apparent in people below 70 years of age whereas it is not statistically significant in people aged 70 and over. Reasons why older individuals do not also manifest higher CVD-related mortality warrant further investigation but may be related to a higher competing risk of non-CVD-related mortality due to frailty, non-CV comorbidities and immunosenescence, such that CVD has relatively minor additional prognostic effect. Similar age-dependent mortality effects have been reported in other studies [6, 7].

### Cardiovascular risk factors

CV risk factors such as hypertension (56%) and diabetes (35%) were more prevalent than established CVD in our in-patient cohort, similar to other UK studies [4]. There are conflicting data regarding the prognostic impact of common CV risk factors in COVID-19. In hospitalised cohorts such as the UK ISARIC study, diabetes had a marginal independent effect on mortality risk, similar to our findings [4]. The majority of patients in ISARIC had uncomplicated diabetes and hypertension was not assessed. In the population-based OpenSAFELY study, diabetes was independently associated with a higher mortality, whereas hypertension was not independently associated with mortality [6]. Another large UK population-based study reported a 1.8-fold higher mortality risk for patients with type 2 diabetes after adjustment for relevant factors [26]. These divergent findings may be reconciled by considering that mortality rates in population-based studies reflect the risk of infection as well as risk of mortality once infected. For example, higher mortality risk associated with diabetes in population-based studies was suggested to be partly related to the level of glycaemic control [7]. We hypothesise that there may be an association between diabetes and infection risk. Previous studies have demonstrated that individuals with diabetes are at increased risk of serious infections [27] and poor glycaemic control has been associated with serious infections and hospital admission [28], although this association has not yet directly been shown for SARS-CoV-2. Nevertheless, even if patients with diabetes are more likely to be admitted, the current study and previous reports [4] suggest that mortality in this group could to a significant degree relate to the co-existence of CVD or other complications.

It should be acknowledged, however, that there may be interactions among CV risk factors and other variables that affect mortality risk e.g., ethnicity [7] and the effective treatment of risk factors [7].

### Cardiovascular complications

A greater mortality risk associated with pre-existing CVD as compared to CV risk factors without CVD raises questions about the potential mechanisms underlying the higher risk. It has been proposed by several authors that endothelial dysfunction may be a major contributor to severe COVID-19 [29, 30]. Accordingly, pre-existing endothelial dysfunction may increase the likelihood of developing severe endothelial and vascular impairment with COVID-19. Such a mechanism would not readily explain the differential risk between established CVD and CV risk factors since both conditions are associated with endothelial dysfunction (and the majority of patients with CVD have CV risk factors). An alternative possibility is that patients with pre-existing established CVD are more prone to develop further cardiac injury and dysfunction which, in combination with pulmonary and right heart problems that represent the major manifestations of severe COVID-19, leads to life-threatening illness.

To explore this possibility, we analysed CV complications. The use of semi-automated pipelines to capture all clinician-diagnosed CV events minimised selection and indication bias. With this, our data demonstrate a high frequency of CV events overall (25%), with the majority (73%) representing arrhythmias, mostly atrial fibrillation, with rates comparable to smaller studies [5, 31, 32]. Other complications included acute MI and acute heart failure, with few clinically-diagnosed cases of myocarditis (0.7%).

Patients with pre-existing CVD had higher rates of CV complications than those with CV risk factors without CVD or patients without either CVD or CV risk factors. They also had higher rates of VTE but, whereas CVD was an independent predictor for incident CV complications, it was not a predictor of VTE. Importantly, we found that a high proportion of COVID-19 related CV complications (mainly cardiac) represent exacerbated or destabilised pre-existing CVD, rather than new presentations. Taken together, these findings suggest that the detrimental impact of pre-existing CVD on COVID-19 severity and mortality may be mediated mainly by increased cardiac problems rather than systemic vascular abnormalities such as VTE. In support of this idea, we found that myocardial injury as assessed by troponin elevation was most prevalent in patients with pre-existing CVD and strongly associated with mortality.

Currently the mechanism of thromboembolic risk in COVID-19 remains unclear. Potential mechanisms that have been suggested include vascular endothelial dysfunction, abnormal complement and coagulant pathway activation, and abnormal platelet activation [33]. The fact that there was no independent association between new-onset VTE and established CVD may partly reflect higher rates of antiplatelet or anticoagulant therapy in this group. Another confounding factor could be undiagnosed VTE [10]. Ongoing clinical trials examining anticoagulation strategies and additional pathophysiology studies will provide further insights into this question.

The high incidence of cardiac arrhythmias, mostly atrial fibrillation, observed in this study may have multiple precipitants, such as myocardial ischaemia, increased sympathetic tone, inflammation (systemic as well as myocardial), and electrolyte imbalance. Our finding of a low incidence of myocarditis is consistent with several other reports and a recent review of autopsy cases [34, 35].

### Clinical implications

Our finding that a large proportion of CV complications represented destabilised pre-existing CVD, supports the importance of identifying CVD in patients presenting to hospital with COVID-19 (including new diagnoses) and maintaining evidence-based CV care, alongside disease-specific treatment for COVID-19, including, for example, continuation of ACE inhibitors or ARBs in individuals with an indication [16]. The high incidence of arrhythmias may warrant more systematic electrocardiographic screening of hospitalised patients, particularly as the detection of new-onset atrial fibrillation is an indication for anticoagulation to reduce the risk of stroke and systemic thromboembolism. The low rates of STEMI in our cohort, matches reports of declining admission rates for STEMI during the pandemic [36], suggesting that clinical deterioration and CV complications in patients with COVID-19 may be less frequently due to STEMI. Additionally, given the age and comorbidity profile of patients hospitalised with severe COVID-19, this population has a high risk of type 2 myocardial infarction (i.e., a mismatch between oxygen supply and demand, without acute atherothrombotic plaque disruption [24]), in the presence or absence of underlying CAD. Since this may have implications for triage and treatment protocols, a low threshold for biomarker (troponin) assessment in patients with pre-existing CVD should be considered.

### Limitations

Our analysis was limited to individuals who required hospital admission and is therefore only generalisable to this population. This was a retrospective study of prospectively entered data in the EHR. Although this study assessed CV risk factors and established CVD as separate entities, a limitation of this approach is that there is a continuum and that individuals with risk factors may have undiagnosed CVD. Nevertheless, the presence of overt diagnosed CVD does appear to distinguish this group in terms of outcomes. During the early stages of the pandemic, echocardiography and coronary angiography were only performed in selected cases in keeping with recommendations to avoid unnecessary cardiac imaging (in order to reduce transmission of the virus, protect healthcare professionals, and conserve personal protective equipment [37]). A small minority of patients were still in hospital and were censored at the study end-date (2.1%). However, a sensitivity analysis in patients who were either discharged or died revealed similar findings (Additional file 3: Fig. S6). Our selection of cardiovascular risk factors was based on those with highest prevalence, most reliably reported, and to further explore findings in preceding large UK population studies [4, 7]. We did not have robust information regarding dyslipidaemia, however a sensitivity analysis reclassifying patients prescribed statin therapy (with no known CVD) as RF-CFD, as a crude measure of hyperlipidaemia or high CV risk, showed similar effect estimates to the main analysis. In addition, there was a significant amount of missing data for BMI, however effect estimates were robust in sensitivity analyses accounting for BMI and obesity, including obesity as a RF-CVD. We considered this approach more appropriate than multiple imputation, because underweight and overweight individuals, may be more likely to have their BMI recorded, thus contradicting the required missing at random assumption [38].

Finally, our multivariable analyses adjusted for patient characteristics and the presence or absence of several comorbidities, however measures of control (e.g., blood pressure control for hypertension or HbA1c for diabetes) were not assessed and may impact the risk of death. Additionally, we cannot exclude residual confounding due to unmeasured non-cardiac comorbidities, such as malignancy, hepatic or non-vascular neurological diseases.

## Conclusions

Among patients hospitalised with COVID-19, pre-existing established CVD appears to be a more important contributor to in-hospital mortality than CV risk factors without co-existent CVD, particularly in patients below the age of 70 years. This enhanced risk may be driven, at least in part, by a higher incidence of cardiac complications and myocardial injury in patients with pre-existing CVD whereas VTE appears less important. Optimal management of pre-existing CVD may serve to modify outcomes related to COVID-19 in this group. In addition, heightened vigilance for arrhythmias and myocardial injury should be considered for patients with pre-existing CVD, to enable early detection and intervention where needed.

## Supplementary Information

**Additional file 1.** Supplemental Methods.

**Additional file 2.** Supplemental Table 1.

**Additional file 3.** Supplemental Figures.

## Data Availability

The authors declare that all data supporting the findings of this study are available within the article (and its supplementary information files). Individual participant data will not be made available due to confidentiality regulations.

## Abbreviations

ACEI: Angiotensin converting enzyme inhibitors
aHR: Adjusted hazard ratio
ARB: Angiotensin receptor blockers
ARDS: Acute respiratory distress syndrome
BMI: Body mass index
CKD: Chronic kidney disease
COPD: Chronic obstructive pulmonary disease
COVID-19: Coronavirus disease 2019
CV: Cardiovascular
CVD: Cardiovascular disease
DPP4: Dipeptidyl-peptidase 4
DVT: Deep vein thrombosis
ESC: European Society of Cardiology
EHR: Electronic health record
GLP1: Glucagon-like peptide 1
HR: Hazard ratio
hs-cTnT: High sensitivity cardiac troponin T
ICU: Intensive care unit
IQR: Interquartlie range (25th–75th percentile)
NHS: National Health Service
MI: Myocardial infarction
NLP: Natural language processing
PE: Pulmonary embolism
RF: Cardiovascular risk factors
RF-CVD: Cardiovascular risk factors without established cardiovascular disease
RT-PCR: Reverse transcriptase polymerase chain reaction
SARS-CoV-2: Severe acute respiratory distress syndrome-coronavirus-2
SGLT2: Sodium glucose co-transporter-2
STEMI: S-T elevation myocardial infarction
SD: Standard deviation
VTE: Venous thromboembolism
