# Supplementary material for: Pre-existing cardiovascular disease rather than cardiovascular risk factors drives mortality in COVID-19
Source: BMC Cardiovasc Disord. 2021 Jul 3;21:327. doi: 10.1186/s12872-021-02137-9 (PMC8254437; doi:10.1186/s12872-021-02137-9)
Supplement: Supplementary file 1 — Additional file 1. Supplemental Methods. [file 12872_2021_2137_MOESM1_ESM.docx]

**Supplemental methods**

*Classification of Cardiovascular Diseases (CVD) and complications of COVID-19*

Myocardial infarction, heart failure, myocarditis, pericarditis were defined according to clinician diagnoses in accordance with internationally recognised guidelines(1-4). Patients with specific cardiomyopathies (e.g., amyloidosis, sarcoidosis and hypertrophic cardiomyopathy) who had a concomitant clinical diagnosis of heart failure documented in the EHR (based on symptoms and signs) were categorised as heart failure. Since the prevalence of specific cardiomyopathies was low, they were not analysed separately. Valvular heart disease refers to ‘severe’ grade disease (i.e., not including mild and moderate disease), as defined by European Society of Cardiology guidelines(5). A pre-existing or new diagnosis of cardiac arrhythmia was defined by the SNOMED CT UK extension parent term “Clinical Arrhythmia” and all daughter terms, and therefore encompasses a wide spectrum of cardiac arrhythmias, including a specific term for atrial fibrillation (which we also report separately). Atrial fibrillation including paroxysmal, persistent and longstanding persistent classifications. Endocarditis was defined according to international guidelines.(6)

Acute myocardial infarction, myocarditis, pericarditis and endocarditis were defined as complications of COVID-19 if a clinician-diagnosis was documented during the hospital admission for COVID-19, based on clinical features, biomarker elevation and where relevant, diagnostic imaging, including echocardiography and coronary angiography, consistent with guideline-directed diagnostic criteria(1, 3, 4, 6). Acute heart failure complicating COVID-19 was defined as a clinician-made diagnosis of acute left or right ventricular failure e.g. acute pulmonary or peripheral oedema, based on clinical criteria and, where available, echocardiography. An exacerbation or decompensation of arrhythmia (including atrial fibrillation) was defined by new or worsening symptoms, haemodynamic instability or poor heart rate control, as documented by the treating physician.

*Cardiovascular drug therapy*

CV drug therapy at the time of admission was recorded including Angiotensin-Converting Enzyme Inhibitors (ACEI), Angiotensin Receptor Blockers (ARB), aldosterone antagonists, beta-blockers, calcium channel antagonists, loop diuretic agents, statins, antiplatelet and anticoagulant therapy.

**Supplemental References**

1. Thygesen K, Alpert JS, Jaffe AS, Chaitman BR, Bax JJ, Morrow DA, et al. Fourth Universal Definition of Myocardial Infarction (2018). Circulation. 2018;138(20):e618-e51.

2. Ponikowski P, Voors AA, Anker SD, Bueno H, Cleland JG, Coats AJ, et al. 2016 ESC Guidelines for the diagnosis and treatment of acute and chronic heart failure: The Task Force for the diagnosis and treatment of acute and chronic heart failure of the European Society of Cardiology (ESC)Developed with the special contribution of the Heart Failure Association (HFA) of the ESC. Eur Heart J. 2016;37(27):2129-200.

3. Caforio AL, Pankuweit S, Arbustini E, Basso C, Gimeno-Blanes J, Felix SB, et al. Current state of knowledge on aetiology, diagnosis, management, and therapy of myocarditis: a position statement of the European Society of Cardiology Working Group on Myocardial and Pericardial Diseases. Eur Heart J. 2013;34(33):2636-48, 48a-48d.

4. Adler Y, Charron P, Imazio M, Badano L, Baron-Esquivias G, Bogaert J, et al. 2015 ESC Guidelines for the diagnosis and management of pericardial diseases: The Task Force for the Diagnosis and Management of Pericardial Diseases of the European Society of Cardiology (ESC)Endorsed by: The European Association for Cardio-Thoracic Surgery (EACTS). Eur Heart J. 2015;36(42):2921-64.

5. Baumgartner H, Falk V, Bax JJ, De Bonis M, Hamm C, Holm PJ, et al. 2017 ESC/EACTS Guidelines for the management of valvular heart disease. Eur Heart J. 2017;38(36):2739-91.

6. Habib G, Lancellotti P, Antunes MJ, Bongiorni MG, Casalta JP, Del Zotti F, et al. 2015 ESC Guidelines for the management of infective endocarditis: The Task Force for the Management of Infective Endocarditis of the European Society of Cardiology (ESC). Endorsed by: European Association for Cardio-Thoracic Surgery (EACTS), the European Association of Nuclear Medicine (EANM). Eur Heart J. 2015;36(44):3075-128.
