# Supplementary material for: Pre-existing cardiovascular disease rather than cardiovascular risk factors drives mortality in COVID-19
Source: BMC Cardiovasc Disord. 2021 Jul 3;21:327. doi: 10.1186/s12872-021-02137-9 (PMC8254437; doi:10.1186/s12872-021-02137-9)
Supplement: Supplementary file 2 — Additional file 2. Supplemental Table 1. [file 12872_2021_2137_MOESM2_ESM.docx]

**Supplemental Table 1. Clinical observations and laboratory results**

|  | **Total**  **N=1721** | **No CVD or**  **CV risk factors**  **N=484**  **(28.1%)** | **RF-CVD**  **N=888**  **(51.6%)** | **CVD**  **N=349**  **(20.3%)** | **p-value** |
| --- | --- | --- | --- | --- | --- |
| **Observations** |  |  |  |  |  |
| Temperature, ºC | 37.0 ± 0.02 | 37.1 ± 0.04 | 37.1 ± 0.02 | 36.8 ± 0.03 | <0.001 |
| Heart rate, /min | 85.7 ± 0.7 | 88.3 ± 2.2 | 84.9 ± 0.6 | 83.9 ± 0.9 | 0.058 |
| Systolic blood pressure, mmHg | 127.0 ± 0.7 | 124.2 ± 1.6 | 129.1 ± 0.8 | 125.4 ± 1.3 | 0.003 |
| Diastolic blood pressure, mmHg | 72.6 ± 0.5 | 72.7 ± 0.8 | 73.7 ± 0.9 | 69.8 ± 0.8 | 0.017 |
| Respiratory rate, /min | 20.5 ± 0.1 | 20.4 ± 0.3 | 20.8 ± 0.2 | 20.0 ± 0.3 | 0.085 |
| Oxygen saturation, % | 96.0 ± 0.1 | 96.0 ± 0.3 | 96.0 ± 0.1 | 96.2 ± 0.1 | 0.649 |
| **Laboratory results** |  |  |  |  |  |
| CRP, mg/L | 97.4 ± 2.2 | 87.4 ± 4.3 | 107.9 ± 84.6 | 84.6 ± 4.3 | <0.001 |
| White cell count, x10^9^/L | 9.0 ± 0.2 | 9.5 ± 0.5 | 8.6 ± 0.2 | 9.2 ± 0.4 | 0.130 |
| Lymphocytes, x10^9^/L | 1.4 ± 0.1 | 1.6 ± 0.2 | 1.3 ± 0.2 | 1.3 ± 0.2 | 0.438 |
| Neutrophils, x10^9^/L | 6.9 ± 0.1 | 7.2 ± 0.3 | 6.7 ± 0.1 | 7.1 ± 0.3 | 0.196 |
| Platelets, x10^9^/L | 234.6 ± 2.5 | 238.2 ± 5.1 | 234.3 ± 3.3 | 230.4 ± 5.8 | 0.571 |
| Sodium, mmol/L | 137.8 ± 0.2 | 138.0 ± 0.3 | 137.7 ± 0.2 | 137.7 ± 0.4 | 0.659 |
| Urea, mmol/L | 9.7 ± 0.2 | 6.6 ± 0.3 | 10.6 ± 0.3 | 11.7 ± 0.4 | <0.001 |
| eGFR, mL/min/m^2^ | 60.1 ± 0.6 | 72.1 ± 1.0 | 57.3 ± 0.9 | 50.7 ± 1.4 | <0.001 |
| Albumin, g/L | 36.9 ± 0.1 | 38.0 ± 36.7 | 36.7 ± 0.2 | 35.6 ± 0.3 | <0.001 |
| ALP | 99.4 ± 2.2 | 96.1 ± 4.3 | 94.3 ± 3.0 | 117.3 ± 5.0 | <0.001 |
| Bilirubin | 12.3 ± 0.6 | 14.4 ± 1.7 | 10.9 ± 0.5 | 12.9 ± 0.9 | 0.031 |
| High sensitivity cardiac troponin T, ng/L |  |  |  |  |  |
| Baseline* | 21 (9-50) | 11 (5-31) | 22 (11-50) | 43 (19-76) | <0.001 |
| N | 552 | 145 | 308 | 99 |  |
| N (%) elevated** | 349 (63.2) | 63 (43.4) | 203 (65.9) | 83 (83.8) |  |
| Peak | 36 (16-85) | 27 (10-71) | 35 (16-84) | 48 (23-93) | <0.001 |
| N | 742 | 186 | 409 | 147 |  |
| N (%) elevated** | 567 (76.4) | 123 (66.1) | 311 (76.0) | 133 (90.5) |  |

Data reported as mean ± SEM (median [IQR] for high sensitivity cardiac troponin T)

CRP, C-reactive protein; CV, cardiovascular; CVD, cardiovascular disease; eGFR, estimated glomerular filtration rate; ALP, alkaline phosphatase; RF-CVD, cardiovascular risk factors without established CVD.

*Within 24 hours of admission

**Greater than 14ng/L
