# Supplementary material for: Pre-existing cardiovascular disease rather than cardiovascular risk factors drives mortality in COVID-19
Source: BMC Cardiovasc Disord. 2021 Jul 3;21:327. doi: 10.1186/s12872-021-02137-9 (PMC8254437; doi:10.1186/s12872-021-02137-9)
Supplement: Supplementary file 3 — Additional file 3. Supplemental Figures. [file 12872_2021_2137_MOESM3_ESM.docx]

**Supplemental Figures**

|  |  | Page |
| --- | --- | --- |
| Supplemental Figure 1 | Prevalence of pre-existing cardiovascular disease and risk factors by A) age and B) ethnic group | 2 |
| Supplemental Figure 2 | Risk of in-hospital mortality stratified by age and cardiovascular risk group with patients on statin therapy and no known cardiovascular disease included in the RF-CVD group (n=64 patients reclassified). | 3 |
| Supplemental Figure 3 | Risk of in-hospital mortality stratified by age and cardiovascular risk group in patients with BMI data available, adjusting for BMI | 4 |
| Supplemental Figure 4 | Risk of in-hospital mortality stratified by age and cardiovascular risk group in patients with BMI data available, adjusting for obesity (BMI≥30kg/m^2^). | 5 |
| Supplemental Figure 5 | Risk of in-hospital mortality stratified by age and cardiovascular risk group in patients with BMI data available. Obesity included as a CV risk factor if no known cardiovascular disease (i.e., 83 patients with BMI≥30kg/m^2^ reclassified as RF-CVD). | 6 |
| Supplemental Figure 6 | Risk of in-hospital mortality stratified by age and cardiovascular risk group in patients who were discharged or died (i.e. excluding current in-patients). | 7 |
| Supplemental Figure 7 | Incidence of COVID-19 related cardiovascular complications in patients with pre-existing established cardiovascular disease. | 8 |
| Supplemental Figure 8 | Risk of in-hospital mortality stratified by age, cardiovascular risk group and myocardial injury A. Any troponin elevation (hs-cTnT>14ng/L). B. Troponin elevation >10x upper limit of normal (hs-cTnT>140ng/L) | 9 |

**Supplemental Figure 1. Prevalence of pre-existing cardiovascular disease and risk factors**

1. **By age group**

**
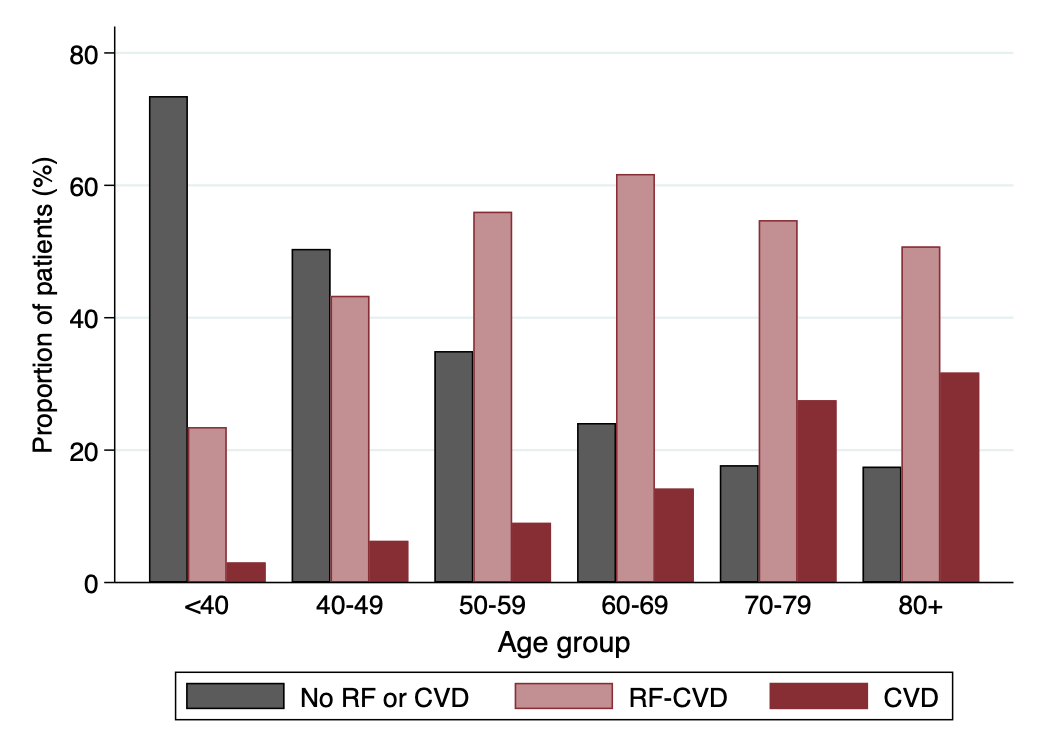
**

1. **By ethnic group**


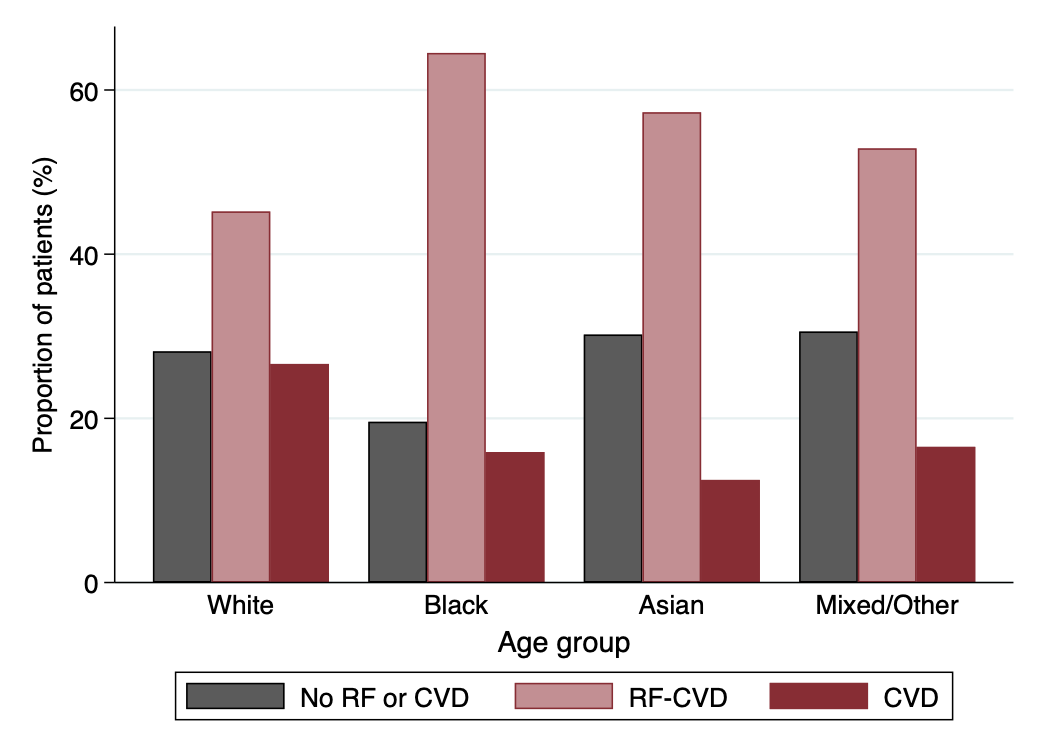


Bars represent the proportion of patients (%) within each age or ethnic group.

CVD, cardiovascular disease; RF, cardiovascular risk factors; RF-CVD, cardiovascular risk factors without established cardiovascular disease;

**Supplemental Figure 2. Risk of in-hospital mortality stratified by age and cardiovascular risk group with patients on statin therapy and no known cardiovascular disease included in the RF-CVD group (n=64 patients reclassified).**

**A. Age <70 years (n=492)**

**B. Age 70 years and over (n=539)**

aHR, adjusted hazard ratio; CVD, cardiovascular disease; RF, cardiovascular risk factors; RF-CVD, cardiovascular risk factors without established CVD.

Model 1 adjusted for age, sex and ethnicity.

Model 2 adjusted for age, sex, ethnicity, BMI, non-cardiac comorbidities (asthma, COPD, chronic renal failure, pulmonary embolism, DVT) and medications (ACEI or ARB, aldosterone receptor antagonists, beta-blockers, calcium channel blockers, loop diuretics, statins, anticoagulants, antiplatelet agents, metformin, sulphonylureas, SGLT2 inhibitors, DPP4 inhibitors, thiazolidinediones, GLP1 receptor agonists, insulin).

**Supplemental Figure 3. Risk of in-hospital mortality stratified by age and cardiovascular risk group in patients with BMI data available, adjusting for BMI.**

**A. Age <70 years (n=492)**

**B. Age 70 years and over (n=539)**

aHR, adjusted hazard ratio; CVD, cardiovascular disease; RF, cardiovascular risk factors; RF-CVD, cardiovascular risk factors without established CVD.

Model 1 adjusted for age, sex and ethnicity.

Model 2 adjusted for age, sex, ethnicity, BMI, non-cardiac comorbidities (asthma, COPD, chronic renal failure, pulmonary embolism, DVT) and medications (ACEI or ARB, aldosterone receptor antagonists, beta-blockers, calcium channel blockers, loop diuretics, statins, anticoagulants, antiplatelet agents, metformin, sulphonylureas, SGLT2 inhibitors, DPP4 inhibitors, thiazolidinediones, GLP1 receptor agonists, insulin).

**Supplemental Figure 4. Risk of in-hospital mortality stratified by age and cardiovascular risk group in patients with BMI data available, adjusting for obesity (BMI≥30kg/m^2^)**

**A. Age <70 years (n=492)**

**B. Age 70 years and over (n=539)**

aHR, adjusted hazard ratio; CVD, cardiovascular disease; RF, cardiovascular risk factors; RF-CVD, cardiovascular risk factors without established CVD.

Model 1 adjusted for age, sex and ethnicity.

Model 2 adjusted for age, sex, ethnicity, obesityI, non-cardiac comorbidities (asthma, COPD, chronic renal failure, pulmonary embolism, DVT) and medications (ACEI or ARB, aldosterone receptor antagonists, beta-blockers, calcium channel blockers, loop diuretics, statins, anticoagulants, antiplatelet agents, metformin, sulphonylureas, SGLT2 inhibitors, DPP4 inhibitors, thiazolidinediones, GLP1 receptor agonists, insulin).

**Supplemental Figure 5. Risk of in-hospital mortality stratified by age and cardiovascular risk group in patients with BMI data available. Obesity included as a CV risk factor (i.e., patients with BMI≥30kg/m^2^ included in the RF-CVD group).**

**A. Age <70 years (n=492)**

**B. Age 70 years and over (n=539)**

aHR, adjusted hazard ratio; CVD, cardiovascular disease; RF, cardiovascular risk factors; RF-CVD, cardiovascular risk factors without established CVD.

Model 1 adjusted for age, sex and ethnicity.

Model 2 adjusted for age, sex, ethnicity, non-cardiac comorbidities (asthma, COPD, chronic renal failure, pulmonary embolism, DVT) and medications (ACEI or ARB, aldosterone receptor antagonists, beta-blockers, calcium channel blockers, loop diuretics, statins, anticoagulants, antiplatelet agents, metformin, sulphonylureas, SGLT2 inhibitors, DPP4 inhibitors, thiazolidinediones, GLP1 receptor agonists, insulin).

**Supplemental Figure 6. Risk of in-hospital mortality stratified by age and cardiovascular risk group in patients who were discharged or died (i.e. excluding current in-patients).**

**A. Age <70 years (n=799)** ****

**B. Age 70 years and over (n=885)**

aHR, adjusted hazard ratio; CVD, cardiovascular disease; RF, cardiovascular risk factors; RF-CVD, cardiovascular risk factors without established CVD.

Model 1 adjusted for age, sex and ethnicity.

Model 2 adjusted for age, sex, ethnicity, BMI, non-cardiac comorbidities (asthma, COPD, chronic renal failure, pulmonary embolism, DVT) and medications (ACEI or ARB, aldosterone receptor antagonists, beta-blockers, calcium channel blockers, loop diuretics, statins, anticoagulants, antiplatelet agents, metformin, sulphonylureas, SGLT2 inhibitors, DPP4 inhibitors, thiazolidinediones, GLP1 receptor agonists, insulin).

**Supplemental Figure 7. Incidence of COVID-19 related cardiovascular complications in patients with pre-existing established cardiovascular disease.**


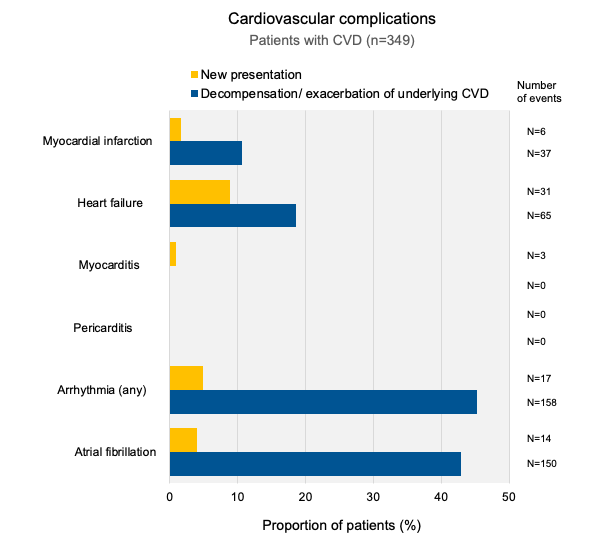


CVD denotes established cardiovascular disease

Bars represent the proportion (%) of patients with CVD experiencing that complication. The blue bars represent events occurring in patients with a previous (or underlying chronic) diagnosis of the respective condition, e.g. myocardial infarction occurring in an individual with a previous myocardial infarction, and acute decompensated heart failure occurring in an individual with known chronic heart failure. Exacerbation/decompensation of arrhythmia-related complications (including atrial fibrillation) refer to new or worsening symptoms, haemodynamic instability or poor heart rate control. Note that patients can experience more than 1 complication. Total number of cases displayed at the end of each bar.

**Supplemental Figure 8. Risk of in-hospital mortality stratified by age, cardiovascular risk group and myocardial injury (n=742 patients)**

**A. Any troponin elevation (hs-cTnT>14ng/L)**

**
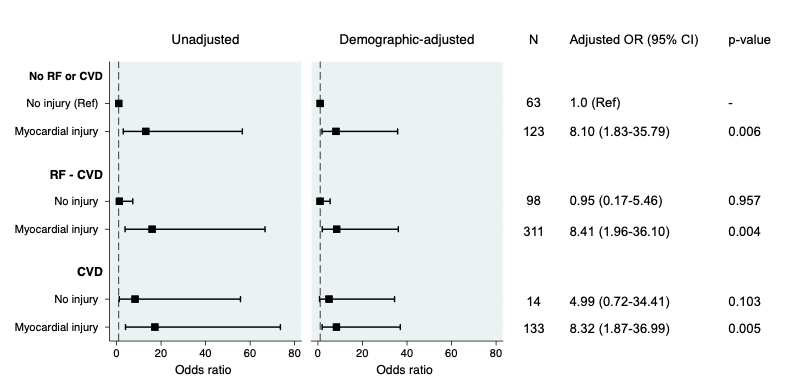
**

**B. Troponin elevation >10x upper limit of normal (hs-cTnT>140ng/L)**


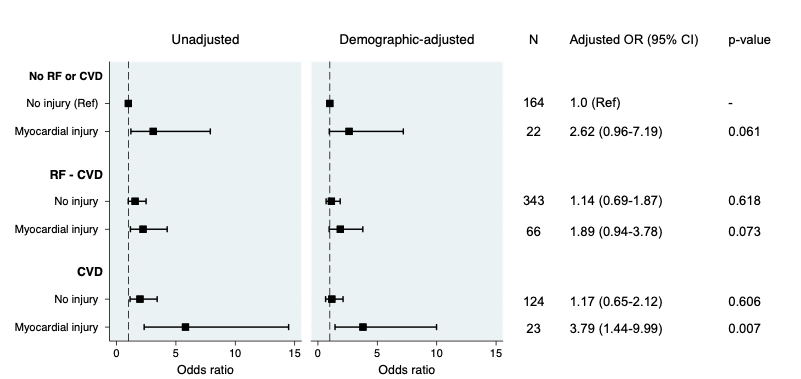


aOR, adjusted odds ratio; CVD, cardiovascular disease; RF, cardiovascular risk factors; RF-CVD, cardiovascular risk factors without established CVD.

Demographic-adjusted model: adjusted for age, sex and ethnicity.
